# Impact of hemodynamic goal-directed resuscitation on mortality in adult critically ill patients: a systematic review and meta-analysis

**DOI:** 10.1007/s10877-017-0032-0

**Published:** 2017-06-08

**Authors:** Maria Cronhjort, Olof Wall, Erik Nyberg, Ruifeng Zeng, Christer Svensen, Johan Mårtensson, Eva Joelsson-Alm

**Affiliations:** 1 Department of Clinical Science and Education, Södersjukhuset, Karolinska Institutet, Stockholm, Sweden; 2 Unit of Anaesthesiology and Intensive Care, Södersjukhuset, Stockholm, Sweden; 3 The Second Hospital and Yuying Children’s Hospital, Wenzhou Medical College, Wenzhou, China; 4 Department of Anesthesiology, The University of Texas Medical Branch UTMB Health, John Sealy Hospital, Galveston, USA; 5 Section of Anaesthesia and Intensive Care Medicine, Department of Physiology and Pharmacology, Karolinska Institutet, Solna, Sweden; 6 Department of Intensive Care, Austin Hospital, Melbourne, VIC Australia

**Keywords:** Critical care, Hemodynamic monitoring, Protocol, Fluid therapy, Meta-analysis, Mortality

## Abstract

The effect of hemodynamic optimization in critically ill patients has been challenged in recent years. The aim of the meta-analysis was to evaluate if a protocolized intervention based on the result of hemodynamic monitoring reduces mortality in critically ill patients. We performed a systematic review and meta-analysis according to the Cochrane Handbook for Systematic Reviews of Interventions. The study was registered in the PROSPERO database (CRD42015019539). Randomized controlled trials published in English, reporting studies on adult patients treated in an intensive care unit, emergency department or equivalent level of care were included. Interventions had to be protocolized and based on results from hemodynamic measurements, defined as cardiac output, stroke volume, stroke volume variation, oxygen delivery, and central venous-or mixed venous oxygenation. The control group had to be treated without any structured intervention based on the parameters mentioned above, however, monitoring by central venous pressure measurements was allowed. Out of 998 screened papers, thirteen met the inclusion criteria. A total of 3323 patients were enrolled in the six trials with low risk of bias (ROB). The mortality was 22.4% (374/1671 patients) in the intervention group and 22.9% (378/1652 patients) in the control group, OR 0.94 with a 95% CI of 0.73–1.22. We found no statistically significant reduction in mortality from hemodynamic optimization using hemodynamic monitoring in combination with a structured algorithm. The number of high quality trials evaluating the effect of protocolized hemodynamic management directed towards a meaningful treatment goal in critically ill patients in comparison to standard of care treatment is too low to prove or exclude a reduction in mortality.

**Electronic supplementary material:**

The online version of this article (doi:10.1007/s10877-017-0032-0) contains supplementary material, which is available to authorized users.

## Introduction

Hemodynamic optimization in critically ill patients is performed very differently in intensive care settings worldwide [1, 2]. The obvious reason to give a patient fluid would be to improve cardiac output and organ perfusion. The challenge is to identify patients who might benefit from fluid, since only 50% of patients are considered to be fluid responders in a general intensive care population [3]. In a recent global observational study of fluid challenges in critically ill patients, only 22% of the patients were evaluated with dynamic indices for fluid responsiveness, such as Stroke Volume Variation (SVV), Pulse Pressure Variation (PPV), or a change in Stroke Volume (SV) after a Passive Leg Raising (PLR) test. Regardless of the response to the first fluid bolus approximately 50% of the patients received a second bolus of fluid [4]. Furthermore, only 8.2% of fluid boluses given to patients in septic shock in French ICUs were monitored with continuous measurements of cardiac output [5]. To assess fluid responsiveness many clinicians use clinical examination, pulse rate, blood pressure, central venous pressure (CVP) or urinary production. However, such static measures are poor markers of fluid responsiveness [6–9]. Logically, the use of dynamic parameters such as real-time changes in SV or cardiac output should provide more accurate measures of the physiological response to fluid therapy. Since monitoring alone cannot be expected to improve outcome, it is important to combine the monitoring with a structured intervention.

Goal-directed fluid therapy (GDT) is widely used in the perioperative setting and has been shown to reduce mortality [10] and surgical complications in small trials [11–13]. However, this concept was recently challenged in a large randomized controlled trial [14], showing no reduction in mortality or morbidity. Yet, a subsequent meta-analysis demonstrated that GDT was associated with fewer infections and shorter duration of hospital stay. Another recent meta-analysis showed that GDT based on dynamic parameters reduced morbidity in the perioperative setting [15].

Critically ill patients react differently to fluids compared to patients who present for elective surgery. For patients with sepsis the problem of vasodilatation and increased capillary permeability reduces the peak effect and duration of the volume expanding effect of intravenous fluids. The concept of Early Goal-Directed Therapy (EGDT) was introduced by Rivers et al. 2001 as a specific treatment protocol utilized for the first 6 h in patients with severe sepsis or septic shock [16]. However, recently three large clinical trials randomizing >4000 septic patients to early goal directed therapy (EGDT) did not show any reduction in mortality from the protocolized approach [17–19]. Angus et al. performed a meta-analysis of the effect of EGDT in sepsis [20], where there was no reduction in mortality in the EGDT-groups. Furthermore, there is a need to evaluate the effect of hemodynamic optimization in all critically ill patients in the ICU and in patients where treatment algorithms have been used longer than 6 h. The aim of this meta-analysis was to evaluate if hemodynamic monitoring combined with a structured and extended treatment plan before or during the stay in the ICU reduces mortality in critically ill patients.

## Materials and methods

We performed a systematic review and meta-analysis according to the Cochrane Handbook for Systematic Reviews of Interventions [21]. The Preferred Reporting Items for Systematic Review and Meta-analysis Protocols (PRISMA-P) statement was followed in setting up and reporting the meta-analysis [22]. The quality of evidence was assessed using the GRADE system [23]. The study was registered in the PROSPERO database (CRD42015019539).

### Type of studies

We only included randomized clinical trials (RCT) published in English.

### Inclusion criteria

To define the meta-analysis, we used the Cochrane acronym PICO (Participants, Interventions, Comparators, and Outcomes).

#### Participants

Participants were adult patients treated at an ICU, emergency department or corresponding level of care.

#### Intervention

Interventions had to be protocolized and based on results from hemodynamic measurements, defined as cardiac output (CO), SV, SVV, oxygen delivery, central venous oxygenation (ScvO2) or mixed venous oxygenation (SvO2).

#### Comparators

The control group had to be treated with standard of care, without any structured intervention based on the parameters mentioned above, however, monitoring by CVP measurements were allowed.

#### Outcome

Primary outcome was all-cause mortality at any time point. Positive fluid balance and weight gain were defined as secondary endpoints.

### Exclusion criteria

Exclusion criteria were perioperative, pediatric and animal studies. If the intervention was initiated before or in the operating room, the intervention was defined as perioperative.

### Search strategy

We searched the Pub Med, Embase and CENTRAL databases for articles using the following search terms: [(“intensive care” OR “intensive care units” OR “ICU” OR critically ill OR critical illness OR emergency service OR emergency department) AND (cardiovascular agents OR fluid therapy)]. The search was performed 18/12/2014 with an updated search 04/01/2016. The search strategy was adapted until all known articles were included in the search. Full search strategies are available in the Supplementary Appendix1.

### Selection of studies

The searches were performed with the assistance of a librarian and all titles and abstracts were first independently reviewed for relevancy by two persons in the team of reviewers (MC, EJA, OW, EN, and RZ). In addition, references lists and expert opinions were reviewed for further relevant studies. Full-texts of articles that could meet inclusion criteria were read by two reviewers and either included or excluded. If there were different opinions two more reviewers examined the article and consensus was reached after a joint discussion in the team.

### Data extraction

Two reviewers per article used a data collection form customized from the standardized Cochrane Collaborative form to extract data from each article. All relevant information such as study size, type of monitoring, intervention and control used, primary and secondary outcomes were registered using this form.

### Quality assessment

Risk of bias (ROB) was assessed independently by two reviewers using the Cochrane Collaborative Tool for Risk of Bias Assessment [21]. It is not possible to blind the treating staff from what hemodynamic algorithm is being used. Thus we moderated the intention in the pre-specified plan (PROSPERO CRD42015019539) to disregard non-blinding in the ROB assessment.

### Statistical methods

Outcome measurement was expressed as pooled OR with a 95% CI, presented as a forest plot. We used the χ^2^ statistic to assess statistical heterogeneity of treatment effect, where a p-value < 0.10 was interpreted as evidence of heterogeneity. We also used the I^2^ statistic to assess the impact of statistical heterogeneity on the treatment effect. I^2^ 0–40% was interpreted as if the inconsistency might not be important. We used the Mantel–Haenszel random effects model even if significant statistical heterogeneity was not present This was because the assumptions of the fixed effect model were not fulfilled (the same direction and size of effect in all studies). We also had large clinical heterogeneity. τ^2^ was used to describe the estimate of between study variance in the random effects analyses. Sensitivity analyses were performed for arbitrary decisions; with and without trials with more than one domain with unclear or high risk of bias and with/without a validated hemodynamic measurement tool. We performed a Trial Sequential Analysis (TSA) to calculate the required sample size to be able to exclude a random finding of no effect [24, 25]. The Trial Sequential Software (Copenhagen Trial Unit, Copenhagen, Denmark) was used for the TSA [26]. We considered results as statistically significant with a two-sided p-value <0.05. The statistical analyses were performed by RevMan 5.3 software, The Nordic Cochrane Centre, Copenhagen, Denmark.

## Results

**Fig. 1:**
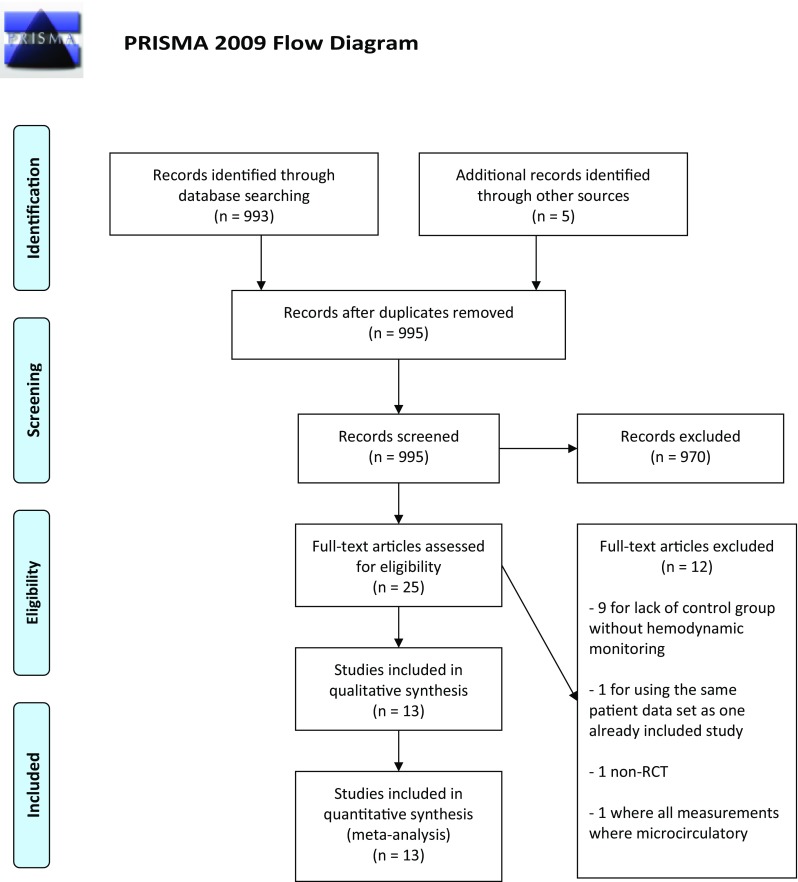
PRISMA flow diagram Fig. 1 Legend: PRISMA flow diagram on literature search and study selection

Out of 998 screened papers, thirteen met the inclusion criteria (Fig. 1). A total of 6850 patients were enrolled in the included studies. There were 3323 patients in the six trials with low ROB. No studies reported the secondary endpoints of positive fluid balance or body weight.

### Study characteristics

**Fig. 2:**
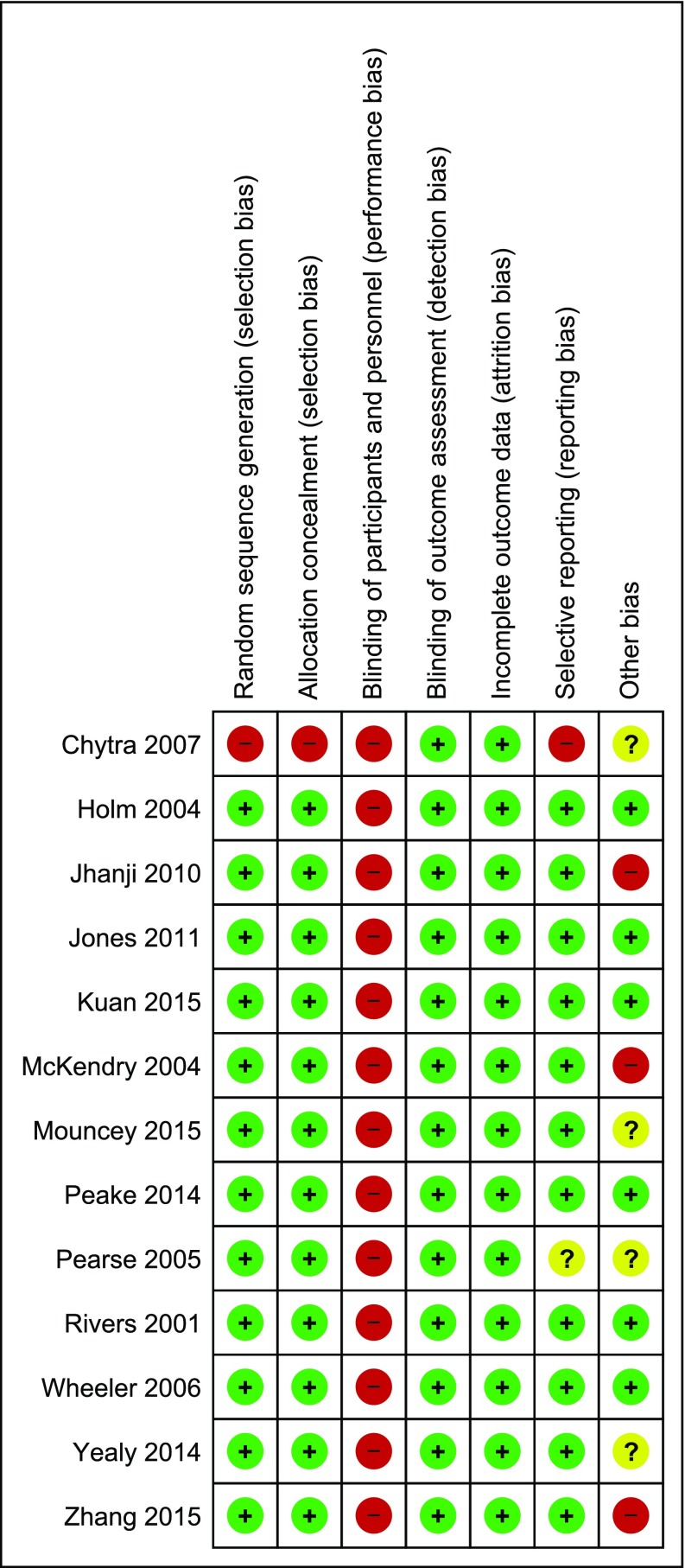
Risk of bias assessment for included studies Fig. 2 Legend: Assessment of validity of included studies according to the cochrane collaborative tool for risk of bias assessment. Low risk of bias +, high risk of bias −, unclear risk of bias?

The trials that were included and their characteristics are described in Table 1 [16–19, 27–35]. All were randomized clinical open-label trials. Five were EGDT trials [16–19, 31] which recruited 4735 patients and eight trials were not EGDT protocol studies which recruited 2115 patients. Six trials were assessed as overall low ROB, five studies were unclear or high risk in one other domain, and two studies were unclear or high in two or more other domains (Fig. 2). Low inclusion to screening rate without explanations lead to the exclusion of two recent large multicentre EGDT trials [17, 19]. Detailed ROB assessments are available in Supplementary Appendix 2.

**Table 1:** Description of included trials

Author and year (ref)	Country	Population	Multi or single centre	Severity of illness score	Type of monitoring in the intervention group	Hemodynamic protocol	Type of control protocol	Duration of protocol	No. of patients	Outcome
Chytra [30]	Czech Republic	ICU trauma patients	Single centre	APACHE II Intervention 20 (16–21) Control 18 (12–23)	Oesophageal doppler	250 ml colloid bolus if FTc < 0,35 s until SV increased < 10%	MAP and CVP	12 h	162	Hospital mortality
Holm [28]	Germany	Burn unit patients	Single centre	ABSI Intervention 8.6 Control 8.9	Trans-pulmonary thermodilution	Fluid boluses if ITBVI ≤800 ml/m^2^ or CI <3.5 L min × m^2^. Limited fluids if EVLWI >10 ml/kg	Treatment according to Baxter formula	48 h	50	Hospital mortality
Jhanji [34]	Great Britain	Abdominal surgery patients	Single centre	ASA score Intervention 2 (2–3) Control 2 (2–3)	Lithium indicator dilution	Colloid bolus to achieve 10% increased SV ± dopexamine infusion	Colloid boluses for CVP increase of 2 mm Hg	8 h	135	Hospital mortality within 28 days
Jones [31]	USA	Septic shock patients	Multi centre	SOFA Intervention 6.6 ± 3.5 Control 6.7 ± 3.6	ScvO2	Crystalloid boluses to achieve CVP >8 mmHg and MAP >65 mmHg. RBC transfusion or dobutamine to achieve ScvO2 ≥70%	Lactate clearance	6 h	300	Hospital mortality
Kuan [27]	Singapore	Severe sepsis/ septic shock patients	Single centre	SOFA Intervention 3.6 ± 3.0 Control 3.2 ± 2.1	Bioreactance	Fluid bolus if PLR gave > 10% increase in SVI	MAP, usual care by clinician	3 h or until discharge from ER	122	28 day mortality
McKendry [35]	Great Britain	Cardiac surgery patients	Multi centre	APACHE II Intervention 10 Control 11	Oesophageal doppler	Fluid bolus for 10% SI increase, with nitrates added if SI > 35 ml/m2 and MAP > 70 or adrenaline if SI > 35 ml/m2 and MAP < 70	Usual care	4 h	179	Hospital mortality
Mouncey [17]	Great Britain	Septic shock patients	Multi centre	APACHE II Intervention 20 ± 6.9 Control 19 ± 7.1	ScvO2	Crystalloid boluses to achieve CVP > 8 mmHg. RBC transfusion or dobutamine to achieve ScvO2 ≥70%	Usual care	6 h	1260	90 day mortality
Peake [18]	Australia & New Zealand	Septic shock patients	Multi centre	APACHE II Intervention 15.4 ± 6.5 Control 15.8 ± 6.5	ScvO2	Crystalloid boluses to achieve CVP > 8 mmHg. RBC transfusion or dobutamine to achieve ScvO2 ≥ 70%	Usual care	6 h	1600	90 day mortality
Pearse [29]	Great Britain	ICU high risk surgical patients	Single centre	APACHE II Intervention 9.4 ± 3.9 Control 9.6 ± 4.3	Lithium indicator dilution	Fluid bolus to increase SVI > 10%, Dopexamin to increase DO_2_I ≥ 600 ml/min × m^2^	MAP and CVP	8 h	122	60 day mortality
Rivers [16]	USA	Septic shock patients	Single centre	APACHE II Intervention 20.4 ± 7.4 Control 21.4 ± 6.9	ScvO2	Crystalloid boluses to achieve CVP > 8 mmHg RBC transfusion or dobutamine to achieve ScvO2 ≥70%	MAP and CVP	6 h	267	Hospital mortality
Wheeler [33]	USA	ARDS-patients	Multi centre	APACHE III Intervention 94.7 ± 1.4 Control 93.5 ± 1.4	PAC	Fluid bolus if MAP < 60 mmHg and UOP < 0,5 ml/kg/h or CI <2.5 L min × m^2^ and PAOP <18 mmHg liberal, conservative 12 mmHg	Fluid bolus if MAP <60 mmHg and UOP <0,5 ml/kg/h or mottling and CVP <8 mmHg conservative, 14 mmHg liberal	7 days or until 12 h after extubation	1001	60 day mortality
Yealy [19]	USA	Septic shock patients	Multi centre	APACHE II Intervention 20.8 ± 8.1 Control 20.7 ± 7.5	ScvO2	Crystalloid boluses to achieve CVP > 8 mmHg. RBC transfusion or dobutamine to achieve ScvO2 ≥70%	Heart rate/systolic blood pressure or usual care	6 h	1341	Hospital mortality at 60 days
Zhang [32]	China	Septic shock and/or ARDS-patients	Multi centre	APACHE II Intervention 29 (21–35) Control 24 (17–31)	Transpulmonary thermodilution	Colloid boluses to achieve ITBVI ≥850 ml/min/m^2,^ dobutamine to achieve CI >2.5 L min × m^2^	MAP and CVP	10 days or until 48 h after stabilization	350	28 day mortality

Values are presented as mean ± standard deviation or as median (interquartile range) *APACHE* acute physiology and chronic health evaluation, *ABSI* abbreviated burn severity index *CI* cardiac index, *CVP* central venous pressure, *DO*_*2*_ *I* oxygen delivery indexed to body surface, *EVLWI* extra vascular lung water index, *FTc* flow time corrected, *ITBVI* intra thoracic blood volume index, *MAP* mean arterial pressure, *PAC* pulmonary artery Catheter, *PAOP* pulmonary artery occlusion pressure, *PLR* passive leg raising, *ScvO2* central venous oxygenation, *SOFA* sequential organ failure assessment, *SVI* stroke volume index, *UOP* urinary output

**Fig. 3:**
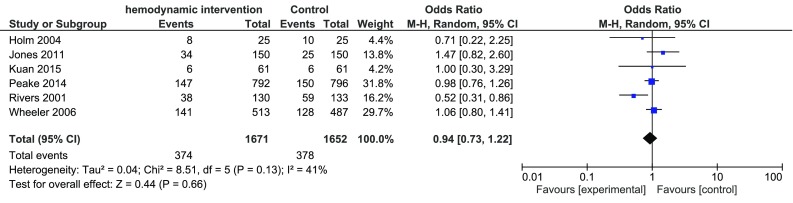
Meta-analysis of effectiveness of hemodynamic monitoring combined with protocolized interventions to reduce mortality, low risk of bias trials Fig. 3 Legend: Meta-analysis of effectiveness of hemodynamic monitoring combined with protocolized interventions to reduce mortality, low risk of bias trials. Weight is the relative contribution of each study to the overall treatment effect (odds risk ratio and 95% confidence interval) on a log scale assuming Mantel–Haenszel random effects model

The analysis including the six trials with low risk of bias [16, 18, 27, 28, 31, 33] resulted in a mortality rate of 22.4% (374/1671 patients) in the intervention group and 22.9% (378/1652 patients) in the control group, OR 0.94 with a 95% CI of 0.73–1.22. Exploring the heterogeneity of treatment effect in the included studies with the χ^2^ statistic showed no evidence of heterogeneity, but according to the I^2^ statistic the percentage of variability of the effect estimate that was due to heterogeneity was large; I^2^ = 41% (Fig. 3).

**Fig. 4:**
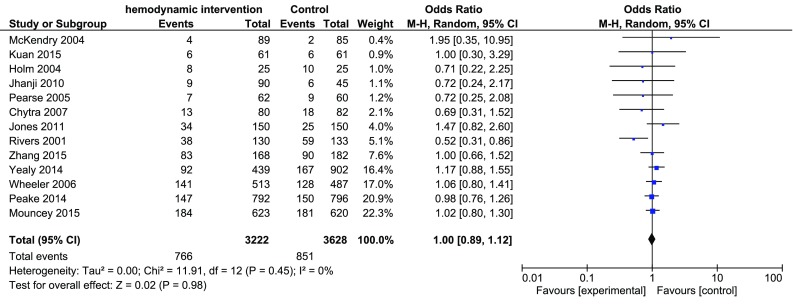
Meta-analysis of effectiveness of hemodynamic monitoring combined with protocolized interventions to reduce mortality, all included trials Fig. 4 Legend: Meta-analysis of effectiveness of hemodynamic monitoring combined with protocolized interventions to reduce mortality, all included trials. Weight is the relative contribution of each study to the overall treatment effect (odds risk ratio and 95% confidence interval) on a log scale assuming Mantel–Haenszel random effects model

In the analysis of all the thirteen included trials the mortality was 23.8% (766/3222 patients) in the intervention group versus 23.5% (851/3628 patients) in the control group with an OR of 1.00 (95% CI 0.89–1.12), see Fig. 4. There was no statistically significant difference in mortality between the groups regardless of whether just the trials with low ROB were analyzed or all trials. However, the TSA showed that a sample size of 17,532 patients would have been needed to be able to exclude that the negative finding was random.

### Results of sensitivity analyses

Analysis of the ten trials with a validated hemodynamic measurement technique, excluding the trials that used bioreactance [27] and esophageal doppler [30, 35], resulted in a mortality of 24.8% (743/2992 patients) in the intervention group and 24.3% (825/3400 patients) in the controls, with an OR of 1.01, 95% CI 0.89–1.13.

The quality of the evidence according to GRADE was considered as moderate (⊕⊕⊕⊝).

## Discussion

We found no statistically significant reduction in mortality from hemodynamic optimization using hemodynamic monitoring in combination with a structured algorithm in the analysis of the six trials with low ROB or in the analysis with all thirteen trials. The sample size of the meta-analysis was too small to exclude that the negative effect was random. There is a paucity of studies looking specifically at hemodynamically guided interventions in the critically ill. Mortality was low in the control group compared to many sepsis trials, which might depend on the mix of patients (including trauma, burn patients and high risk surgical patients as well as septic shock patients) and the time at which mortality was measured (hospital/28 days/90 days). The effect of an intervention on mortality would be expected to be smaller if the mortality in the control group is low. In contrast to our result, there are several meta-analyses that found positive effects of hemodynamic optimization [36, 37] but they mainly included studies that were investigations of mortality before and after introduction of an EGDT protocol. A recent meta-analysis of RCTs by Gu et al. concluded that there is a positive effect of EGDT [38]. They stated that the control group received standard therapy or usual care. However, there were violations of the study protocol since they have included five trials where the control groups were treated according to hemodynamic protocols [39–43]. Moreover, they included trials with high risk of bias in the analysis. Finally, their meta-analysis was conducted before the publication of the Australasian Resuscitation In Sepsis Evaluation (ARISE) [44] and the Protocolized Management in Sepsis (PROMISE) [17] trials which contributed with a substantial number of patients. Our results are consistent with a more recent meta-analysis by Angus et al. limited to septic patients [20]. They included studies that compared EGDT to other hemodynamic protocols or standard of care. They included 13 trials; the same five EGDT trials as in our meta-analysis plus one pediatric [45] and five articles published in Chinese [46–50]. They found no reduction in mortality by EGDT; mortality was 23.2% in the EGDT group compared to 22.4% in the control group, OR 1.01 (95% CI 0.88–1.16). They found only 2 out of 13 studies with low risk of bias, but they included all trials in the analysis. Poeze et al. performed a meta-analysis 2005, using the same hemodynamic variables as in our meta-analysis [10]. They also included studies where two hemodynamic protocols were compared. Consequently, the only overlapping study with our meta-analysis was the Rivers trial [16]. They found no reduction in mortality in patients with septic shock or organ failure.

Our results add to the current knowledge by investigating a broader population cohort without limiting the selection to patients with sepsis. We only included studies with a structured intervention where the controls were treated according to usual care or with simple hemodynamic protocols based on CVP, MAP or lactate or a combination of those parameters. We performed the meta-analysis according to the Cochrane handbook for systematic reviews of interventions and excluded studies with high ROB with the exception of the domain of blinding. Our results stress the lack of studies that evaluate relevant goals for hemodynamic optimization of critically ill patients.

A key question while performing a meta-analysis is the quality of the included studies. Even though we used the structured method of bias assessment according to Cochrane, we found that there were other weaknesses in the trials that were not detected by this method. To investigate if mortality can be reduced by individualized hemodynamic management the monitoring technique should be accurate and precise. During the last decade, several less invasive techniques to measure cardiac output have been introduced [51]. Unfortunately, there are no generally accepted criteria for acceptable agreement that can be applied in the validation of cardiac output measurement techniques. There are criteria suggested by Critchley and Critchley [52], but these are not always followed [53]. The main issue is that there is a constant variation of cardiac output in humans, thus no real precision of measurement can be obtained, At best, estimation of the precision is actually just serial measurements [54]. However, nine of the trials used measurement techniques that are generally accepted as validated in critically ill patients. A sensitivity analysis with these trials showed slightly higher mortality in the intervention group than in the controls, a non-significant result. This neither proved or refuted the hypothesis that it is important to use a validated measurement technique, maybe because the effect of the intervention was small. One trial used a PAC [55], which is considered the gold standard of clinical cardiac output measurement. Two studies used transpulmonary thermodilution [28, 32] which has been validated against PAC and been found to be sufficiently accurate for clinical purposes [56]. Two studies used a lithium dilution technique [29, 34], which is another validated dilution technique [57]. Five trials used continuous ScvO2 to estimate the need for fluids, inotropic agents and blood transfusions according to the EGDT protocol. This method has been validated against intermittent ScvO2 measurements with blood gas analyzers and been found to have excellent accuracy and acceptable precision [58]. Two trials used measurement techniques which are not as well validated in critically ill patients. One of those trials used thoracic bioreactance [27], where there are conflicting results from validation. A meta-analysis has shown that precision is too poor for clinical use [59] while others deemed the accuracy and precision acceptable for clinical use in most situations [60]. Esophageal doppler was used in two trials [30, 35]. It is a technique where measurements depend entirely on correct positioning of the probe which can be cumbersome in the critical care setting. It is mainly used perioperatively, whereas validation in the intensive care setting has shown significant underestimation of CO, especially in women [61].

For a hemodynamic algorithm to be able to reduce mortality the intervention must be directed towards a meaningful treatment goal. Dynamic parameters have been shown to reflect fluid responsiveness better than static parameters and the use of dynamic parameters are recommended over static parameters by experts in the European Society of Intensive Care [62]. Only one of the included trials used a dynamic parameter; the difference in stroke volume index (SVI) caused by a PLR test [27]. This was a small trial using a less precise CO-measurement (bioreactance) and not dimensioned to show a mortality difference. The three large trials that replicated the original EGDT protocol by Rivers did not show any mortality benefit from the protocol using ScvO2 compared to standard of care. A problem with ScvO2 is that most patients in septic shock would have a hyperdynamic circulation with supranormal ScvO2 values, hence the hemodynamic protocol does not lead to a change of treatment. In a small Dutch study looking at ScvO2 values in patients with septic shock the mean ScvO2 was 74% ± 10. Only 6% had a ScvO2 < 60% [63]. ScvO2 is used as a surrogate measurement for oxygen saturation in mixed venous blood (SvO2), and like SvO2 it depends on Hb, arterial saturation and CO as well as oxygen demand. There is great inter patient variability in ScvO2, since it reflects the attempts of the organs to extract the demanded oxygen [64]. The EGDT trials also used CVP to guide the initial fluid therapy, which we excluded from this meta-analysis because it has been repeatedly proven that CVP is of limited value in predicting fluid responsiveness [9]. In a recent meta-analysis with individual patient data a low CVP of 2–8 mmHg had a positive predictive value for fluid responsiveness from 56 to 65% [6].

To answer the question if a critically ill patient ought to be hemodynamically monitored the controls must receive standard care without hemodynamic monitoring. In this meta-analysis we chose to disregard CVP as a hemodynamic monitoring tool, due to the poor correlation with fluid responsiveness.

There may be several ways of understanding the lack of evidence of benefit from individualized hemodynamic management although it has been a key feature of intensive care ever since the introduction of the PAC [65]. Either the task of designing and performing a clinical trial with an algorithm that will suit all critically ill patients is too challenging. Or maybe clinical judgment based on many different parameters as temperature and color of the skin, mental impairment, urinary output and blood pressure is a composite variable that seldom can be reflected by a single hemodynamic measurement. Perhaps we have still to find relevant goals for hemodynamic optimization of critically ill patients. Another explanation might be the heterogeneity in critically ill patients. It is possible that only a subgroup of the most severely ill patients might benefit from structured hemodynamic optimization. That hemodynamic optimization is unnecessary could also be a possible conclusion, but there are not enough studies to prove this.

Our meta-analysis has several strengths. It was planned according to the PRISMA guidelines, registered at PROSPERO and performed according to the Cochrane methodology. A broad search strategy was used and a broad spectrum of studies was included, not only large EGDT trials. We further applied the clinical fact that most critically ill patients are not treated by advanced hemodynamic protocols [5] and investigated the results of studies where the controls were treated according to usual care or with simple hemodynamic protocols based on CVP, MAP or lactate or a combination of those parameters.

Limitations of our study are the clinical diversity in patients, protocols and hemodynamic measurements. The fact that different trials had different timeframes to look at mortality as endpoints is another limitation, as well as that non-English language studies were not included. We did not include observational studies which limits the capacity to evaluate side effects of protocolized hemodynamic management. We also excluded trials that did not evaluate mortality, fluid balance or weight gain.

## Conclusions

This meta-analysis contributes to new knowledge about hemodynamic monitoring combined with structured treatment of critically ill patients in the ICU. In contrast to recent meta-analyses, which only dealt with septic patients, this study also includes studies with other types of critically ill patients. Furthermore, we only included studies in which the intervention group was treated according to a structured plan while the control group was treated according to usual care or with simple hemodynamic protocols based on CVP, MAP or lactate or a combination of those parameters. There are too few high quality trials evaluating the effect of protocolized hemodynamic management directed towards a meaningful treatment goal in critically ill patients in comparison to standard of care treatment to prove or exclude a reduction in mortality.

## Electronic supplementary material

Below is the link to the electronic supplementary material.

Supplementary material 1 (DOCX 15 KB)

Supplementary material 2 (DOCX 28 KB)
