# Supplementary material for: Impact of hemodynamic goal-directed resuscitation on mortality in adult critically ill patients: a systematic review and meta-analysis
Source: J Clin Monit Comput. 2017 Jun 8;32(3):403–14. doi: 10.1007/s10877-017-0032-0 (PMC5943381; doi:10.1007/s10877-017-0032-0)
Supplement: Supplementary file 1 — Supplementary material 1 (DOCX 15 KB) [file 10877_2017_32_MOESM1_ESM.docx]

**Impact of hemodynamic goal-directed resuscitation on mortality in adult critically ill patients: a systematic review and meta- analysis**

Maria Cronhjort^1^, Olof Wall, Erik Nyberg, Ruifeng Zeng, Christer Svensen, Johan Mårtensson, Eva Joelsson-Alm

^1^ Department of Clinical Science and Education, Karolinska Institutet, Unit of Anaesthesiology and Intensive Care, Södersjukhuset, Stockholm, Sweden. Maria.cronhjort@sll.se

**Search strategies**

An initial search was performed 31/12/2014 in Pubmed and Embase. The search terms were ("Intensive Care” OR “intensive care units” OR “ICU”) AND mortality AND hemodynamics AND (cardiovascular agents OR fluid therapy). In Pubmed 403 articles were found and in Embase 113 articles were found.

A complimentary, broader search was performed 04/01/2016:

| **PubMed** **20160104** | | |
| --- | --- | --- |
| 1 | intensive care[MeSH Terms] OR intensive care unit[MeSH Terms]) OR intensive care unit[Title/Abstract]) OR intensive care[Title/Abstract]) OR icu[Title/Abstract] OR (“emergency service, hospital”[MeSH Terms] ) OR emergency department*[ti/ab] OR critical ill*[ti/ab] OR critical illness[Mesh] | 251 334 |
| 2 | Mortality [all fields] | **938 451** |
| 3 | Cardiovascular agents[MeSH] OR Cardiovascular agent*[ti/ab] OR Fluid therapy[MeSH] OR Fluid therapy*[ti/ab] | 249 528 |
| 4 | 2 AND 3 | **21 591** |
| 5 | 1 AND 4 | 1 866 |
| 6 | 5 AND random* [all fields] AND has abstract AND English | 400 |

| **CENTRAL** (**Cochrane Central Register of Controlled Trials) 20160104** (Search manager) | | |
| --- | --- | --- |
| 1 | (Intensive care OR icu OR intensive care unit OR critical ill* OR emergency department OR emergency service) (ti.ab.kw) | 16 347 |
| 2 | Mortality (ti.ab.kw) | 29 245 |
| 3 | (Cardiovascular agents OR Fluid therapy) (ti.ab.kw) | **13 582** |
| 4 | 2 AND 3 | **2 218** |
| 5 | 1 AND 4 | 270 |
| 6 | 5 AND random* | 254 |
| 7 | (6 AND english) NOT (conference OR note) | 186 |

| **Embase 20160104** | | |
| --- | --- | --- |
| 1 | “Intensive care”/exp OR icu:ab,ti OR “intensive care unit” /exp OR critcal illness/exp OR emergency health services/exp | **664 627** |
| 2 | Mortality /exp | **763 369** |
| 3 | ‘Cardiovascular agents’/exp OR ‘Fluid therapy’/exp | **1 598 588** |
| 4 | 2 AND 3 | 74 036 |
| 5 | 1 AND 4 | 15 139 |
| 6 | 5 AND english | 14 244 |
| 7 | 6 AND randomised control trial AND article AND article in press AND adult AND has abstract | 365 |

The complementary search 04/01/2016 resulted in 951 records. Of those, 140 were duplicates and 182 were reviews and meta-analyses.

After removal of records from the search performed 31/12/2014, the complementary search yielded 477 new records. A total of 993 articles were found in the two searches.
