# Supplementary material for: Impact of hemodynamic goal-directed resuscitation on mortality in adult critically ill patients: a systematic review and meta-analysis
Source: J Clin Monit Comput. 2017 Jun 8;32(3):403–14. doi: 10.1007/s10877-017-0032-0 (PMC5943381; doi:10.1007/s10877-017-0032-0)
Supplement: Supplementary file 2 — Supplementary material 2 (DOCX 28 KB) [file 10877_2017_32_MOESM2_ESM.docx]

**Impact of hemodynamic goal-directed resuscitation on mortality in adult critically ill patients: a systematic review and meta- analysis**

Maria Cronhjort^1^, Olof Wall, Erik Nyberg, Ruifeng Zeng, Christer Svensen, Johan Mårtensson, Eva Joelsson-Alm

^1^ Department of Clinical Science and Education, Karolinska Institutet, Unit of Anaesthesiology and Intensive Care, Södersjukhuset, Stockholm, Sweden. maria.cronhjort@sll.se

Risk of Bias Assessment

**Trials with unclear risk of bias (ROB) in one more domain than blinding**: Pearse (29): had unclear ROB (other bias) due to stopping of the trial at interim analysis due to large effect. Yealy (19): unclear ROB (other bias) since many patients were eligible but excluded for study logistic issues. Mouncey (17): unclear ROB (other bias) since many patients were eligible but not included for unclear reasons.

**Trial with high ROB in the domain of other bias:** Zhang (32): Uncertainty of randomization process since there was large differences in baseline characteristics in the allocation groups even though the randomization sequence generation and allocation concealment were properly described. Jhanji (34): The inclusion criteria are not clearly stated. The screening process is not clearly described and a majority of the assessed patients were not included. The compliance to the hemodynamic protocols is not described. McKendry (35): The screening process is not clearly described. Five patients were randomized but not included in the analysis, although it was described that analyses were performed according to intention to treat.

**Trial with high or unclear ROB in several domains**: Chytra (30), high ROB (selection bias) due to pseudo-randomization, high ROB (allocation concealment bias) as patients were allocated to treatment groups according to admission number, high ROB (selective outcome reporting) since organ dysfunction was not reported even though it was a stated primary outcome and unclear ROB(other bias) since it is unclear what impact on outcome there is from a bedside nurse who stays by the patient during the entire study period to ensure correct placement of the esophageal Doppler.

In the large trial by Wheeler (33) comparing CVC to PAC-guided therapy the clinicians did not have to follow the hemodynamic protocol if the patients were unstable. This trial was crucial for the abandoning of PAC-monitoring. This means that we don’t know to what extent the protocols were tested when they were most needed. This is a weakness of the trial, but we did not judge it to be a ROB.
